# Various Doses of Tanezumab in the Management of Chronic Low Back Pain (CLBP): A Pooled Analysis of 4,514 Patients

**DOI:** 10.7759/cureus.46790

**Published:** 2023-10-10

**Authors:** Sophia Tahir, Oman Sadik, Virginia Ezenwa, Chinenye Iguh, Vidhya Ravichandran, Naufin N Ashraf, Erica M O’Connor, Rithika Sayabugari

**Affiliations:** 1 Internal Medicine/Family Medicine, Windsor University School of Medicine, Basseterre, KNA; 2 Family Medicine, Jackson Park Hospital, Chicago, USA; 3 Internal Medicine, Windsor University School of Medicine, Basseterre, KNA; 4 Medicine, Windsor University School of Medicine, Basseterre, KNA; 5 Pediatrics, Sri Muthukumaran Medical College Hospital and Research Institute, Chennai, IND; 6 General Medicine, Gandhi Medical College, Hyderabad, IND

**Keywords:** systematic review and meta-analysis, treating low back pain, chronic low back pain (clbp), chronic pain management, tanezumab

## Abstract

Chronic low back pain (CLBP) is a persistent and debilitating condition characterized by pain and discomfort in the lower back region that lasts more than 12 weeks. This review aims to determine the efficacy and safety of various doses of tanezumab for managing CLBP. The present meta-analysis was reported according to the Preferred Reporting Items for Systematic Reviews and Meta-Analysis (PRISMA) guidelines and the Cochrane Handbook for Systematic Reviews of Intervention standards. We searched multiple databases, including PubMed, Cochrane Library, Excerpta Medica Database (EMBASE), Scopus, and Web of Science, to identify randomized controlled trials comparing tanezumab to placebo or different dosage regimens for CLBP in adult patients. The primary outcome was the mean change in low back pain intensity (LBPI) score baseline to the end of treatment. Secondary outcomes included adverse events and the degree of disability or impairment. A total of six studies were included in the meta-analysis. Analysis of the data showed that tanezumab 5 mg significantly reduced LBPI compared to placebo at all time points (mean deviation (MD) ranging from -0.31 to -0.5). Similarly, tanezumab 10 mg showed a significant reduction in LBPI compared to placebo at all time points (MD ranging from -0.48 to -0.84). However, tanezumab 5 mg showed significantly less reduction of LBPI compared to 10 mg at two, four, eight, and 12 weeks (MD ranging from 0.19 to 0.32). These findings suggest that tanezumab is an effective treatment for CLBP, with 5 mg and 10 mg doses providing clinically meaningful reductions in LBPI.

## Introduction and background

Pain in the lower back that lasts longer than three months is considered chronic and is referred to as chronic low back pain (CLBP) [1]. It can be a debilitating condition that can severely impact a person's quality of life [2]. Chronic low back pain is thought to affect around 20% of the world's adult population [3,4]. It is a complex condition with multifactorial causes, including physical injury, spinal deformities, nerve damage, or degenerative conditions like osteoarthritis [5]. Chronic low back pain can also be associated with a range of symptoms, including stiffness, weakness, numbness, and tingling sensations [6]. The therapy for CLBP may be difficult and often includes a mix of pharmaceutical and non-pharmacological therapies [7]. Commonly used medications for CLBP include non-steroidal anti-inflammatory drugs (NSAIDs), opioids, and muscle relaxants [8]. However, these medications can be associated with various adverse effects, including addiction, dependence, and overdose [9]. Therefore, more effective and safer therapies for CLBP are required.

Tanezumab is a monoclonal antibody that targets nerve growth factor (NGF), a protein involved in the transmission of pain sensations [10]. By inhibiting NGF, tanezumab reduces pain in a variety of illnesses, including CLBP. Tanezumab was primarily created for the treatment of osteoarthritis, but it has also been explored for CLBP, a major global source of disability [11]. The mechanism of action of tanezumab includes limiting the interaction of NGF with its receptors, thereby preventing the transmission of pain signals from peripheral neurons to the central nervous system [12,13]. This action results in a reduction of pain, inflammation, and other associated symptoms of CLBP. Tanezumab is administered through intravenous infusion, and it has been demonstrated to provide a lasting pain-relieving effect [14]. Recently, tanezumab has been administered subcutaneously to increase patient convenience, reduce healthcare costs, and allow self-administration [15].

Previous randomized controlled trials (RCTs) have evaluated tanezumab's efficacy and safety in treating CLBP. These trials have investigated different doses and routes of administration of tanezumab. For instance, one RCT examined the effectiveness of tanezumab (5 mg and 10 mg delivered every eight weeks vs. placebo) in individuals with CLBP [16]. This study indicated that both dosages of tanezumab were significantly more effective than placebo at reducing pain. Another RCT assessed the effectiveness of intravenous tanezumab doses of 5 mg, 10 mg, and 20 mg every eight weeks vs. placebo in individuals with CLBP. The research indicated that tanezumab was significantly related to a decrease in pain compared to placebo [17].

Despite the promising results of these studies, some concerns have been raised about the safety of tanezumab, particularly regarding its potential to increase the risk of joint damage [18]. In a recent trial of tanezumab in osteoarthritis patients, the incidence of adverse events, including joint replacement, was greater in the tanezumab group than in the placebo group [19]. However, it is important to note that the patients in the osteoarthritis study were older and had more comorbidities than the patients in the CLBP studies. Consequently, a meta-analysis of the existing information on the effectiveness and safety of multiple-dose regimens of tanezumab in the treatment of CLBP is required to offer a full and impartial evaluation of the best dosage regimen for tanezumab. This meta-analysis aims to find the most effective and safe dose regimen of tanezumab for treating CLBP by analyzing the results of previous RCTs.

## Review

Methods

This meta-analysis followed the Preferred Reporting Items for Systematic Reviews and Meta-Analyses (PRISMA) and Cochrane Handbook for Systematic Reviews of Interventions standards [20,21].

Search Strategy

A thorough investigation of electronic databases, including Excerpta Medica Database (EMBASE), PubMed, the Cochrane Library, Scopus, and Web of Science, was conducted to identify eligible studies. The search strategy used a combination of medical subject heading (MeSH) terms and keywords related to tanezumab, chronic low back pain, and randomized controlled trials. The search was limited to studies published from inception to May 2023.

Inclusion and Exclusion Criteria

Only RCTs were eligible for inclusion, and these studies had to compare at least two different dosage regimens of tanezumab or a tanezumab dosage regimen with a placebo control group. The studies also had to include adult patients 18 years of age or older with CLBP. Lastly, the studies had to be published in the English language. Non-randomized or observational studies were also excluded, as they were deemed to have lower quality and potentially biased results. Studies that included participants with other types of chronic pain or medical conditions were also excluded to ensure that the results were specific to CLBP. Finally, studies not published in English were also excluded to ensure consistency and ease of analysis.

Study Selection and Data Extraction

Two independent reviewers examined the titles and abstracts of the selected papers to determine their eligibility. Full-text papers were retrieved and assessed for potentially relevant research according to inclusion and exclusion criteria. Disagreements were addressed via dialogue and consensus. Two independent reviewers retrieved data from the included studies using a predefined data extraction form. Each study's author and publication year, study design, sample size, dose regimens for tanezumab, length of therapy, main outcomes, and conclusion were retrieved.

Risk of Bias Assessment

The risk of bias for each included study was independently evaluated by two reviewers using version 2 of the Cochrane Risk of Bias tool [22]. The tool evaluates five domains: bias caused by the randomization technique, bias caused by variations from planned interventions, bias in outcome assessment, bias caused by missing outcome data, and bias in the selection of the reported result.

Data Synthesis and Analysis

Using Review Manager Web ((RevMan Web), Version 5.4.1, The Cochrane Collaboration, 2020), the data from the included studies were analyzed. The main outcome was the mean change in pain ratings from pre-treatment to post-treatment. The pain was evaluated using the low back pain intensity (LBPI) score, a self-reported assessment instrument that evaluates the severity of low back pain in a range of 0 to 10 [23]. Secondary outcomes included adverse events and the degree of disability or impairment. The degree of disability or impairment was assessed by Roland Morris Disability Questionnaire (RMDQ) scores [24].

The mean difference (MD) and 95% confidence interval (CI) were calculated for continuous outcomes. We estimated the risk ratio (RR) and 95% confidence interval for binary outcomes (CI). Using fixed-effects models, meta-analyses were conducted. Heterogeneity was assessed using the P-value, and its extent was assessed by I2. If the data were heterogeneous, we used the random effect model and left one test. Subgroup analyses were conducted regarding doses of tanezumab or follow-up periods.

Results

Study Selection and Characteristics

**Figure 1:**
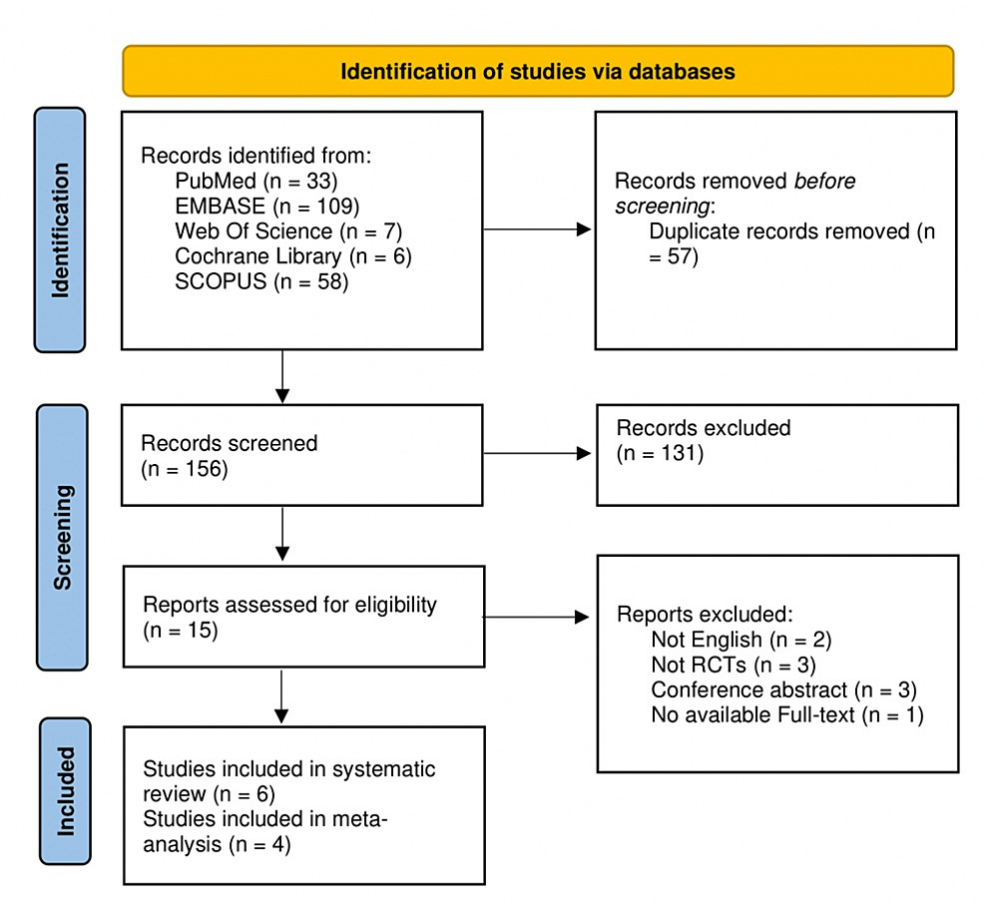
PRISMA flow chart depicting the process of study selection PRISMA: Preferred Reporting Items for Systematic Reviews and Meta-Analyses; EMBASE: Excerpta Medica Database

Figure 1 depicts the selection process.

A total of 213 records were identified from various databases, and 57 duplicate records were removed. After the screening, 15 reports were assessed for eligibility, and six studies were ultimately included in the systematic review [14,16-17,25-27]. Of those six studies, four were included in the meta-analysis [17,25-27]. Three reports were excluded because they were not RCTs, two were not in English, one had no available full text, and three were conference abstracts.

The average age of participants in the studies ranged from 48.4 to 54.3 years, while the percentage of female participants ranged from 40.2% to 60.9%. The majority of participants in the studies were White, with percentages ranging from 75.9% to 82.7%. The reported BMI values ranged from 23.9 kg/m^2^ to 30.3 kg/m^2^. The duration since a CLBP diagnosis ranged from 9.7 to 11.83 years. The reported etiology for CLBP varied among the studies, with degenerative joint disease/osteoarthritis ranging from 13.0% to 44.2%, injury/muscular strain ranging from 0% to 148 (35.5%), degenerative disc disease ranging from 16.0% to 91 (28.4%), and other causes ranging from 1.7% to 45.2%. Further details are shown in Tables 1-2.

**Table 1:** Characteristics of the included studies NR: not reported; NCT: National Clinical Trial number

Study ID	NCT	Tanezumab doses	Follow-up	Primary outcomes	Conclusion
Gimbel et al., 2014 [26]	NCT00924664	10 mg and 20 mg	64 weeks	Change in brief pain inventory short form scores	The tolerability of tanezumab 10 mg was superior to tanezumab 20 mg and could be a useful treatment for chronic low back pain over an extended period.
Katz et al., 2011 [14]	NCT00584870	200 mcg/kg	16 weeks	Change in low back pain intensity	In patients with chronic lower back pain, tanezumab was more effective in reducing pain than both placebo and naproxen, both clinically and statistically.
Kivitz et al., 2013 [17]	NCT00876187	5 mg, 10 mg, and 20 mg	16 weeks	Change in low back pain intensity	Patients with chronic lower back pain who took tanezumab experienced significantly greater improvement in their pain levels, physical function, and overall assessment compared to those who took a placebo or naproxen.
Konno et al., 2021 [25]	NCT02725411	5 mg and 10 mg	80 weeks	Adverse events	They concluded that tanezumab was well-tolerated by most individuals and could potentially alleviate symptoms of chronic lower back pain.
Markman et, al. 2020 [16]	NCT02528253	5 mg and 10 mg	56 weeks	Change in low back pain intensity	Tanezumab at a 10 mg dosage improved pain levels and physical function compared to a placebo in patients with difficult-to-treat chronic lower back pain. However, some joint safety events were associated with tanezumab, including a few cases that required joint replacement.
Markman et al., 2022 [27]	NCT02528253	5 mg and 10 mg	80 weeks	Change in the patient’s global assessment of low back pain	The evidence suggests that tanezumab could be beneficial for some patients with chronic lower back pain compared to a placebo. This is based on measurements of pain, daily function interference, patient assessment of disease status, and treatment satisfaction.

**Table 2:** Baseline characteristics of included studies BMI: body mass index, LBPI: low back pain intensity, RMDQ: Roland-Morris Disability Questionnaire

Study ID	Study arms	Sample	Age, Mean (SD)	Sex, Female, n (%)	White race, n (%)	BMI, Kg2/m^2^, Mean (SD)	Duration since diagnosis of chronic low back pain, years, Mean (SD)	Etiology, n (%)				LBPI, Mean ± SD	RMDQ, Mean ± SD
								Degenerative joint disease/ osteoarthritis	Injury/muscular strain	Degenerative disc disease	others		
Gimbel et al., 2014 [26]	Tanezumab 10 mg	321	53.3 (12.0)	167 (52%)	265 (82.6%)	29.5 (4.9)	11.34 (9.3)	113 (35.2)	114 (35.5)	91 (28.4)	3 (0.9)	-	-
	Tanezumab 20 mg	527	53.2 (11.0)	277 (52.6%)	436 (82.7%)	29.7 (5.2)	11.83 (10.6)	233 (44.2)	148 (28.1)	134 (25.4)	12 (2.3)	-	-
Katz et al., 2011 [14]	Tanezumab 200 mcg/kg	88	49.5 (14.7)	53 (60.2%)	81 (92.0%)	28.8 (4.8)	10 (8.0)	30 (34.1)	21 (23.9)	33 (37.5)	4 (4.5)	6.5 (1.4)	12.3 (4.6)
	Naproxen 500 mg b.i.d	88	52.1 (14.8)	42 (47.7%)	82 (93.2%)	28.6 (4.8)	13 (8.75)	21 (23.9)	20 (22.7)	37 (42.0)	10 (11.4)	6.7 (1.4)	12.4 (4.8)
	Placebo	41	52.2 (15.0)	23 (56.1%)	37 (90.2%)	28.6 (4.4)	9.7 (11.63)	9 (22.0)	10 (24.4)	16 (39.0)	6 (14.6)	6.7 (1.4)	13.7 (5.2)
Kivitz et al., 2013 [17]	Tanezumab 5 mg	232	51.5 (11.7)	115 (49.6%)	187 (80.6%)	29.2 (4.9)	11.3 (8.93)	90 (38.8)	73 (31.5)	64 (27.6)	5 (2.2)	6.6 (1.4)	12.2 (4.9)
	Tanezumab 10 mg	295	52 (11.0)	157 (53.2%)	238 (80.7%)	29.3 (4.9)	12.3 (10.6)	98 (33.2)	109 (36.9)	82 (27.8)	6 (2.0)	6.6 (1.4)	13.0 (5.1)
	Tanezumab 20 mg	295	51.2 (10.2)	165 (55.9%)	227 (76.9%)	29.3 (5.1)	11.2 (9.25)	118 (40.0)	101 (34.2)	66 (22.4)	10 (3.4)	6.7 (1.5)	13.0 (5.0)
	Naproxen 500 mg b.i.d	295	52.6 (11.5)	152 (51.5%)	224 (75.9%)	30.3 (5.0)	10.9 (11.23)	125 (42.4)	96 (32.5)	69 (23.4)	5 (1.7)	6.8 (1.4)	12.9 (4.9)
	Placebo	230	51.2 (11.2)	125 (54.3%)	190 (82.6%)	29.1 (5.2)	11.3 (9.8)	94 (40.9)	73 (31.7)	59 (25.7)	4 (1.7)	6.71 (1.4)	12.8 (4.7)
Konno et al., 2021 [25]	Tanezumab 5 mg	92	53.3( 9.5)	37 (40.2)	-	24.1 (3.9)	-	13 (14.1)	6 (6.5)	32 (34.8)	41 (44.6)	6.7 (1.0)	8.3 (5.0)
	Tanezumab 10 mg	93	52.3 (9.5)	44 (47.3)	-	23.9 (4.2)	-	11 (11.8)	0	40 (43.0)	42 (45.2)	6.8 (1.1)	8.1 (4.9)
	Celecoxib 200 mg	92	54.3 (10.3)	38 (41.3)	-	23.9 (3.6)	-	17 (18.5)	1 (1.1)	38 (41.3)	36 (39.1)	6.7 (1.0)	7.8 (5.0)
Markman et al., 2020 and Markman et al., 2022 [16,27]	Tanezumab 5 mg	407	48.7 (9.5)	248 (60.9)	295 (72.5)	-	11.0 (9.7)	-	-	-	-	7.2 (1.1)	15.0 (5.2)
	Tanezumab 10 mg	407	49.1 (10.3)	218 (53.6)	303 (74.4)	-	10.6 (9.7)	-	-	-	-	7.2 (1.1)	15.0 (4.9)
	Tramadol	602	48.4 (10.3)	339 (56.3)	428 (71.1)	-	11.0 (9.8)	-	-	-	-	7.2 (1.2)	15.1 (5.1)
	Placebo	409	49 (10.7)	236 (57.7)	296 (72.4)	-	11.1 (10.3)	-	-	-	-	7.2 (1.1)	14.8 (5.1)

Risk of Bias Assessment

**Figure 2:**
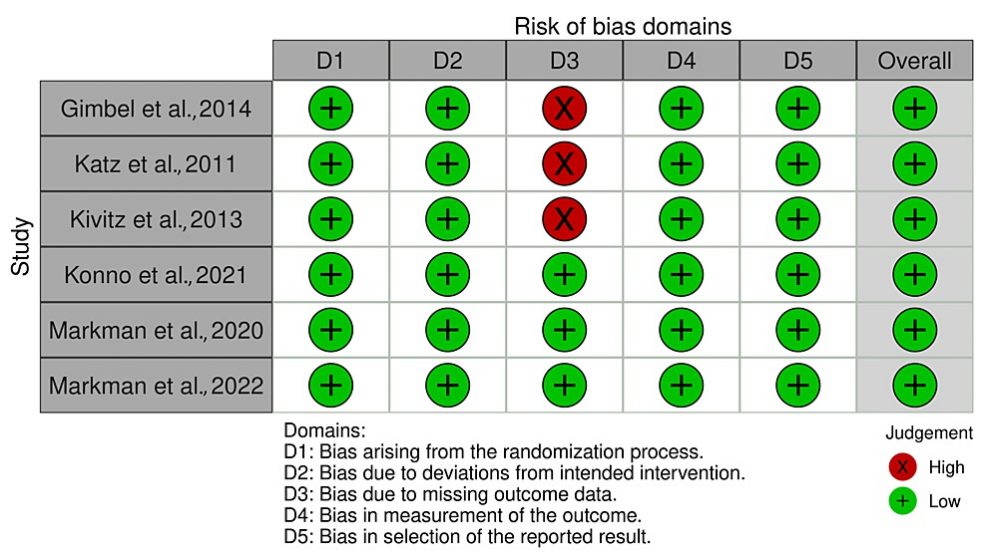
A traffic light plot of the included studies Sources: [14,16-17,25-27]

All studies have a low risk of bias in domains one and two, which relate to randomization and intended interventions, as seen in Figure 2.

Also, all studies have a low risk of bias in domain four (measurement of outcome) and domain five (selection of reported results). However, three studies have a high risk of bias in domain three, which relates to missing outcome data [14,17,26]. 

Primary outcomes

Change in LBPI

**Figure 3:**
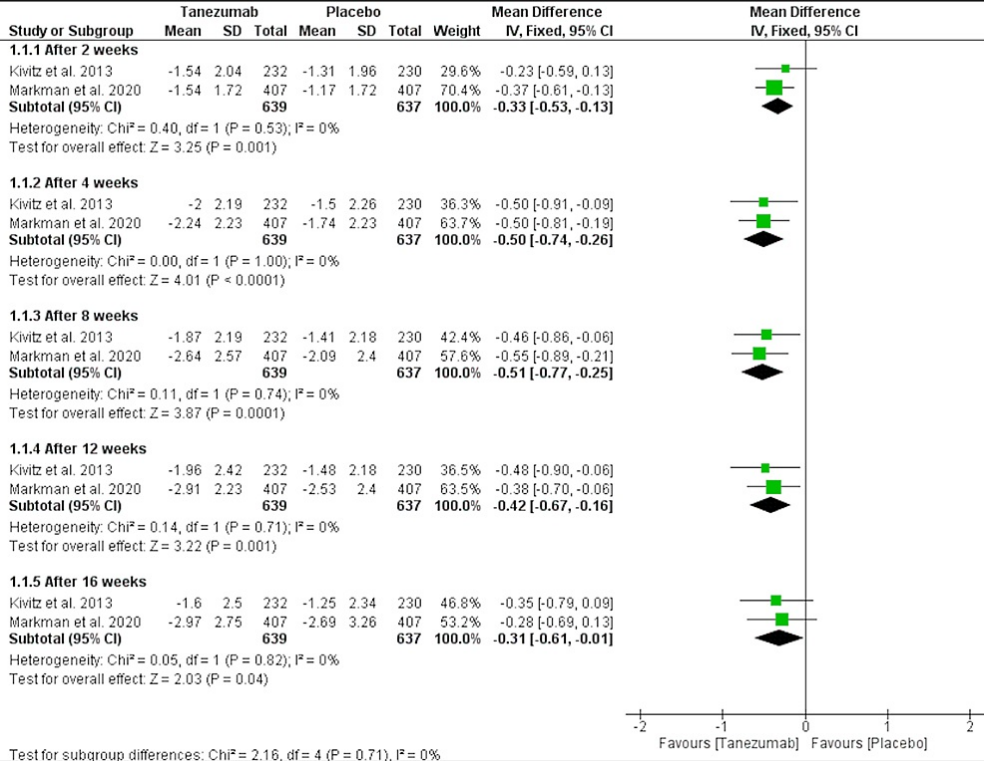
A forest plot comparing the LBPI of tanezumab 5 mg and placebo LBPI: low back pain intensity; CI: confidence interval, SD: standard deviation, IV: inverse variance Sources: [16-17]

Tanezumab 5 mg vs. placebo: Tanezumab 5 mg significantly reduced the LBPI compared to the placebo after 16 weeks [MD = -0.31 (-0.61, -0.01), P = 0.04]. The data were homogenous; I2 was 0, and the P value was 0.82, as shown in Figure 3 and Table 3.

**Table 3:** Pooled analysis of the primary outcomes Bold values under "Effect Estimate" indicate significant results. IV: inverse variance; CI: confidence interval Sources: [16-17,25]

Outcome or Subgroup	Studies	Participants	Statistical Method	Effect Estimate
1. Tanezumab 5 mg Vs. Placebo	2			
1.1. After 2 weeks	2	1276	Mean Difference (IV, Fixed, 95% CI)	-0.33[-0.53,-0.13]
1.2. After 4 weeks	2	1276	Mean Difference (IV, Fixed, 95% CI)	-0.50[-0.74,-0.26]
1.3. After 8 weeks	2	1276	Mean Difference (IV, Fixed, 95% CI)	-0.51[-0.77,-0.25]
1.4. After 12 weeks	2	1276	Mean Difference (IV, Fixed, 95% CI)	-0.42[-0.67,-0.16]
1.5. After 16 weeks	2	1276	Mean Difference (IV, Fixed, 95% CI)	-0.31[-0.61,-0.01]
2. Tanezumab 10 mg Vs. Placebo	2			
2.1. After 2 weeks	2	1277	Mean Difference (IV, Fixed, 95% CI)	-0.48[-0.68,-0.28]
2.2. After 4 weeks	2	1277	Mean Difference (IV, Fixed, 95% CI)	-0.84[-1.09,-0.59]
2.3. After 8 weeks	2	1277	Mean Difference (IV, Fixed, 95% CI)	-0.79[-1.04,-0.53]
2.4. After 12 weeks	2	1277	Mean Difference (IV, Fixed, 95% CI)	-0.68[-0.94,-0.43]
2.5. After 16 weeks	2	1277	Mean Difference (IV, Fixed, 95% CI)	-0.60[-0.89,-0.31]
3. Tanezumab 5 mg Vs. Tanezumab 10mg	3			
3.1. After 2 weeks	3	1464	Mean Difference (IV, Fixed, 95% CI)	0.19[0.01, 0.36]
3.2. After 4 weeks	3	1464	Mean Difference (IV, Fixed, 95% CI)	0.32[0.11, 0.53]
3.3. After 8 weeks	3	1464	Mean Difference (IV, Fixed, 95% CI)	0.30[0.07, 0.54]
3.4. After 12 weeks	3	1464	Mean Difference (IV, Fixed, 95% CI)	0.27[0.03, 0.50]
3.5. After 16 weeks	2	1279	Mean Difference (IV, Fixed, 95% CI)	0.27[-0.01, 0.55]
3.6. After 24 weeks	2	1000	Mean Difference (IV, Fixed, 95% CI)	-0.00[-0.40, 0.40]
3.7. After 32 weeks	2	1000	Mean Difference (IV, Fixed, 95% CI)	-0.17[-0.58, 0.25]
3.8. After 40 weeks	2	1000	Mean Difference (IV, Fixed, 95% CI)	-0.14[-0.57, 0.30]
3.9. After 48 weeks	2	1000	Mean Difference (IV, Fixed, 95% CI)	-0.18[-0.62, 0.25]
3.10. After 56 weeks	2	1000	Mean Difference (IV, Fixed, 95% CI)	-0.13[-0.54, 0.28]

**Figure 4:**
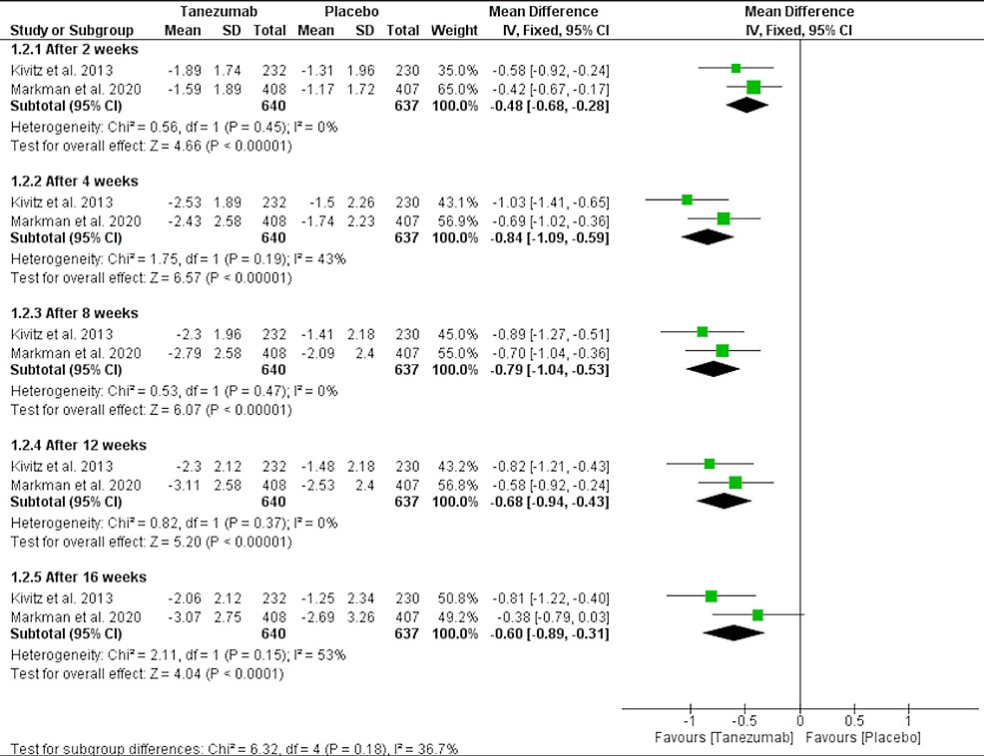
A forest plot comparing LBPI of tanezumab 10 mg and placebo LBPI: low back pain intensity; CI: confidence interval, SD: standard deviation, IV: inverse variance Sources: [16-17]

Tanezumab 10 mg vs. placebo: Tanezumab 10 mg significantly reduced LBPI at 16 weeks compared to placebo [MD = -0.6 (-0.89, -0.31), P < 0.0001], as shown in Figure 4 and Table 3. 

**Figure 5:**
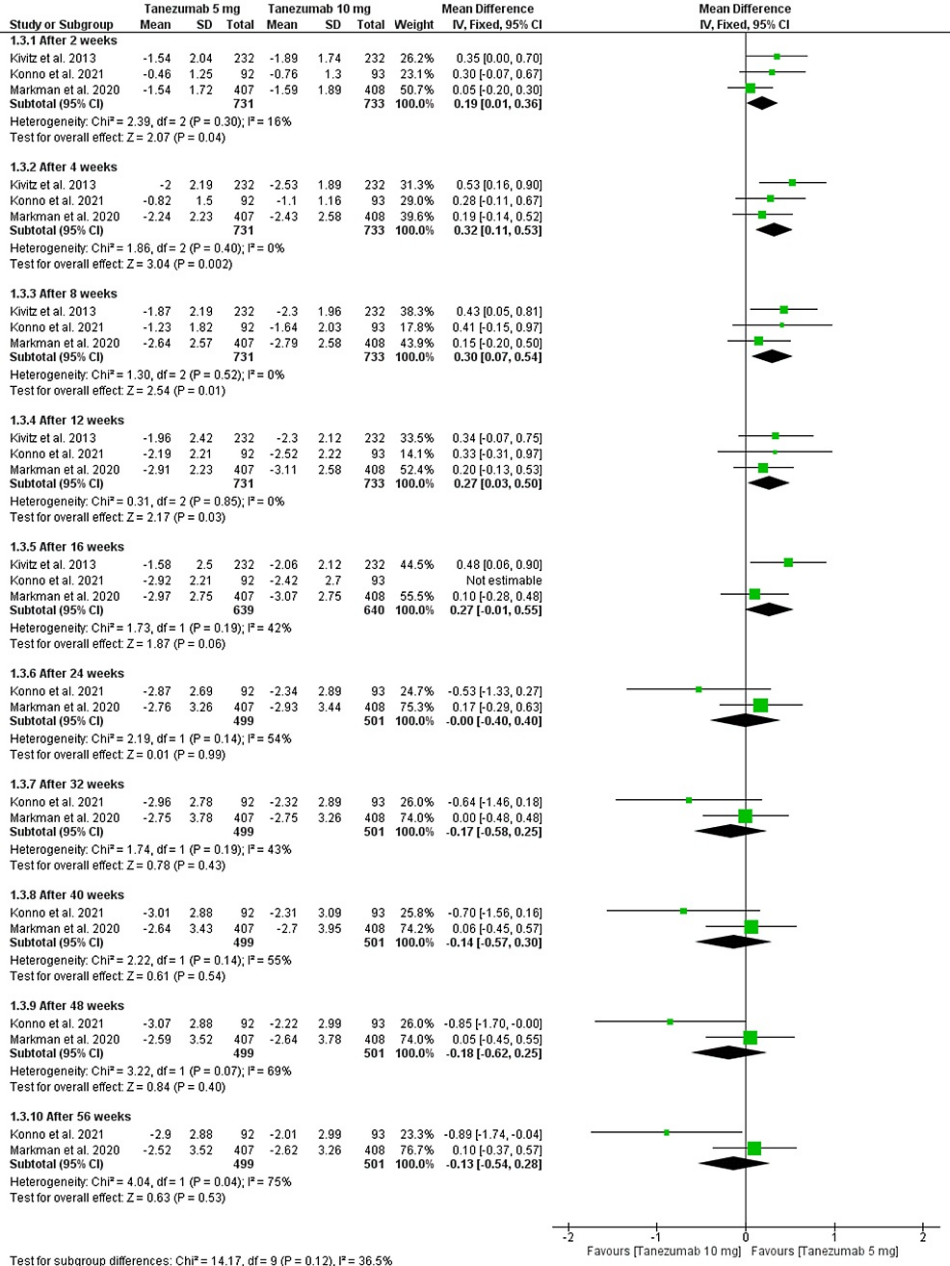
A forest plot comparing LBPI of tanezumab 5 mg and tanezumab 10 mg LBPI: low back pain intensity; CI: confidence interval, SD: standard deviation, IV: inverse variance Sources: [16-17,25]

Tanezumab 5 mg vs. tanezumab 10 mg: Tanezumab 5 mg showed significantly less reduction of LBPI compared to 10 mg at 12 weeks [MD = 0.27 (0.03, 0.5), P = 0.03]. The data were homogenous (P = 0.85, I2 = 0). In contrast, there was an insignificant difference between the two groups after 40 weeks as follows: [MD = -0.14 (-0.57, 0.3), P = 0.54]. The data were homogenous (P = 0.14, I2 = 55%). In addition, there was no significant difference between the two groups after 16 weeks [MD = 0.1 (-0.37, 0.56), P = 0.68], but the data were heterogeneous (P = 0.06, I2 = 64%). This heterogeneity was resolved by excluding Konno et al. 2021 (P = 0.19, I2 = 42%), and the results remained insignificant as follows: [MD = 0.28 (-0.09, 0.65), P = 0.14]. This is evident in Figure 5 and Table 3.

Secondary Outcomes

Change in RMDQ

**Figure 6:**
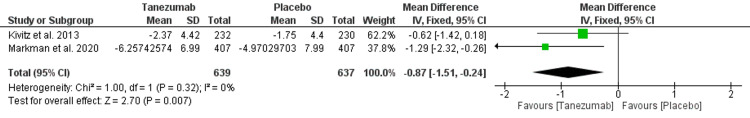
A forest plot of the change in RMDQ for tanezumab 5 mg and placebo LBPI: low back pain intensity; CI: confidence interval, SD: standard deviation, IV: inverse variance; RMDQ: Roland Morris Disability Questionnaire Sources: [16-17]

Tanezumab 5 mg vs. placebo: Tanezumab 5 mg significantly reduced RMDQ compared to placebo after 16 weeks [MD = -0.87 (-1.51, -0.24), P = 0.007], and the data were homogenous (P = 0.32, I2 = 0) as indicated in Figure 6 and Table 4.

**Table 4:** A pooled analysis of secondary outcomes and adverse events Bold values under "Effect Estimate" indicate significant results. M-H: Mantel-Haenszal; CI: confidence interval Sources: [16-17, 25-26]

Outcome or Subgroup	Studies	Participants	Statistical Method	Effect Estimate
1. Tanezumab 5 mg Vs. Placebo	2	1276	Mean Difference (IV, Fixed, 95% CI)	-0.87[-1.51,-0.24]
2. Tanezumab 10 mg Vs. Placebo	2	1277	Mean Difference (IV, Fixed, 95% CI)	-1.53[-2.15,-0.92]
3. Tanezumab 5 mg Vs. Tanezumab 10mg	3			
3.1. After 2 weeks	2	1000	Mean Difference (IV, Fixed, 95% CI)	0.43[-0.17, 1.03]
3.2. After 4 weeks	2	1000	Mean Difference (IV, Fixed, 95% CI)	0.46[-0.14, 1.05]
3.3. After 8 weeks	2	1000	Mean Difference (IV, Fixed, 95% CI)	0.55[-0.13, 1.23]
3.4. After 16 weeks	3	1464	Mean Difference (IV, Fixed, 95% CI)	0.64[0.11, 1.17]
3.5. After 24 weeks	2	1000	Mean Difference (IV, Fixed, 95% CI)	0.09[-0.76, 0.94]
3.6. After 32 weeks	2	1000	Mean Difference (IV, Fixed, 95% CI)	-0.05[-0.89, 0.80]
3.7. After 40 weeks	2	1000	Mean Difference (IV, Fixed, 95% CI)	-0.33[-1.26, 0.61]
3.8. After 48 weeks	2	1000	Mean Difference (IV, Fixed, 95% CI)	-0.32[-1.23, 0.60]
3.9. After 56 weeks	2	1001	Mean Difference (IV, Fixed, 95% CI)	-0.28[-1.21, 0.66]
4. Patients with adverse events	4			
4.1. Tanezumab 5 mg Vs. Placebo	2	1278	Risk Ratio (M-H, Fixed, 95% CI)	1.07[0.96, 1.20]
4.2. Tanezumab 10 mg Vs. Placebo	2	1341	Risk Ratio (M-H, Fixed, 95% CI)	1.12[1.01, 1.24]
4.3. Tanezumab 5 mg Vs. Tanezumab 10 mg	3	1526	Risk Ratio (M-H, Fixed, 95% CI)	0.99[0.90, 1.08]
4.4. Tanezumab 10 mg Vs. Tanezumab 20 mg	2	1438	Risk Ratio (M-H, Fixed, 95% CI)	0.89[0.82, 0.96]
5. Patients with serious adverse events	4			
5.1. Tanezumab 5 mg Vs. Placebo	2	1278	Risk Ratio (M-H, Fixed, 95% CI)	1.11[0.45, 2.71]
5.2. Tanezumab 10 mg Vs. Placebo	2	1341	Risk Ratio (M-H, Fixed, 95% CI)	1.00[0.42, 2.42]
5.3. Tanezumab 5 mg Vs. Tanezumab 10 mg	3	1526	Risk Ratio (M-H, Fixed, 95% CI)	0.78[0.40, 1.53]
5.4. Tanezumab 10 mg Vs. Tanezumab 20 mg	2	1438	Risk Ratio (M-H, Fixed, 95% CI)	1.02[0.57, 1.84]
6. Patients discontinued due to adverse events	4			
6.1. Tanezumab 5 mg Vs. Placebo	2	1278	Risk Ratio (M-H, Fixed, 95% CI)	0.97[0.59, 1.59]
6.2. Tanezumab 10 mg Vs. Placebo	2	1341	Risk Ratio (M-H, Fixed, 95% CI)	1.13[0.71, 1.79]
6.3. Tanezumab 5 mg Vs. Tanezumab 10 mg	3	1526	Risk Ratio (M-H, Fixed, 95% CI)	0.82[0.52, 1.28]
6.4. Tanezumab 10 mg Vs. Tanezumab 20 mg	2	1438	Risk Ratio (M-H, Fixed, 95% CI)	0.76[0.52, 1.12]
7. Arthralgia	4			
7.1. Tanezumab 5 mg Vs. Placebo	2	1278	Risk Ratio (M-H, Fixed, 95% CI)	0.94[0.58, 1.53]
7.2. Tanezumab 10 mg Vs. Placebo	2	1341	Risk Ratio (M-H, Fixed, 95% CI)	1.57[1.02, 2.42]
7.3. Tanezumab 5 mg Vs. Tanezumab 10 mg	3	1526	Risk Ratio (M-H, Fixed, 95% CI)	0.92[0.61, 1.38]
7.4. Tanezumab 10 mg Vs. Tanezumab 20 mg	2	1438	Risk Ratio (M-H, Fixed, 95% CI)	0.86[0.65, 1.15]
8. Parathesia	3			
8.1. Tanezumab 5 mg Vs. Placebo	2	1278	Risk Ratio (M-H, Fixed, 95% CI)	1.88[0.85, 4.19]
8.2. Tanezumab 10 mg Vs. Placebo	2	1341	Risk Ratio (M-H, Fixed, 95% CI)	3.27[1.58, 6.77]
8.3. Tanezumab 5 mg Vs. Tanezumab 10 mg	2	1341	Risk Ratio (M-H, Fixed, 95% CI)	0.58[0.33, 1.03]
8.4. Tanezumab 10 mg Vs. Tanezumab 20 mg	2	1438	Risk Ratio (M-H, Fixed, 95% CI)	0.74[0.54, 1.02]

**Figure 7:**
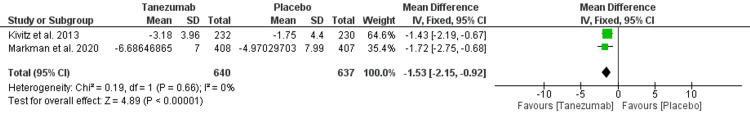
A forest plot of the change in RMDQ for tanezumab 10 mg and placebo LBPI: low back pain intensity; CI: confidence interval, SD: standard deviation, IV: inverse variance; RMDQ: Roland Morris Disability Questionnaire Sources: [16-17]

Tanezumab 10 mg vs. placebo: While tanezumab 10 mg significantly reduced RMDQ compared to placebo after 16 weeks [MD = -1.53 (-2.15, -0.92), P < 0.00001], the data were homogenous (P = 0.66, I2 = 0), as seen in Figure 7 and Table 4.

**Figure 8:**
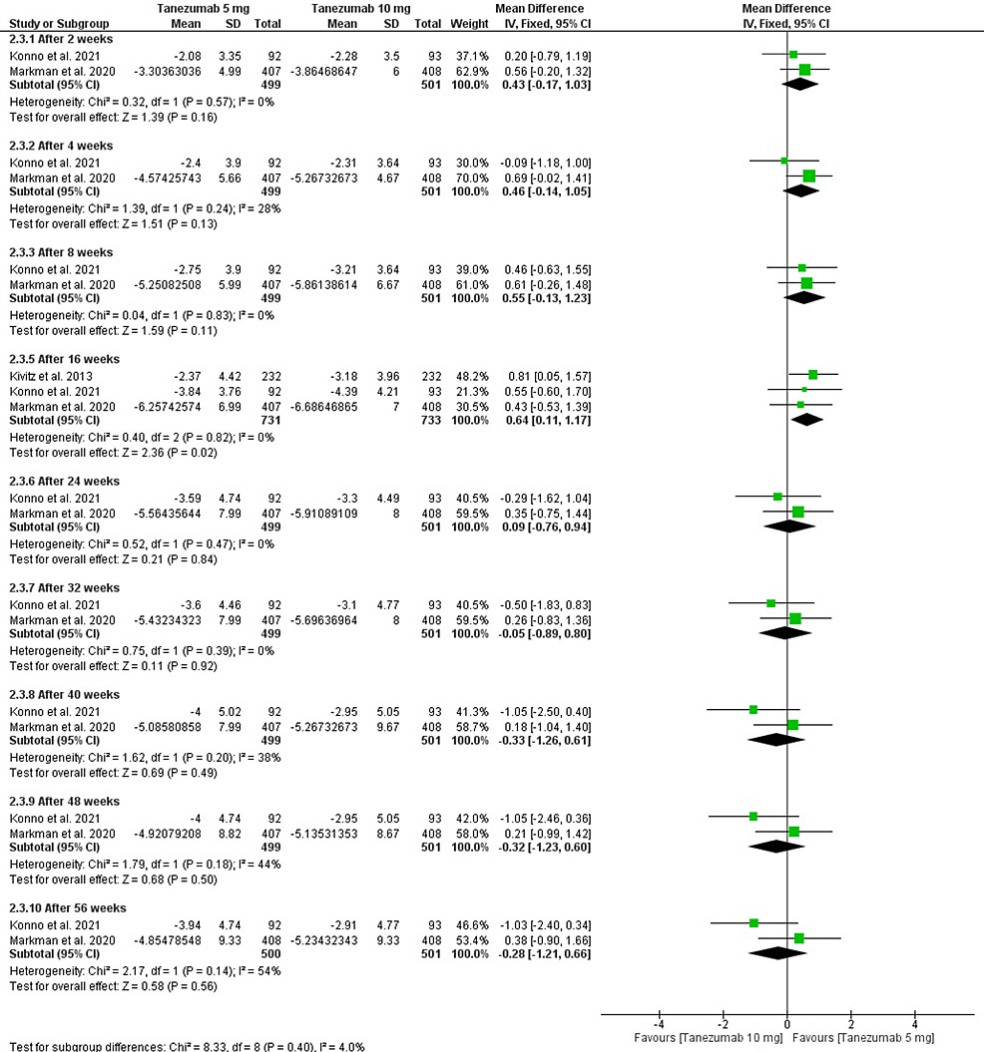
A forest plot of the change in RMDQ for tanezumab 5 mg and tanezumab 10 mg LBPI: low back pain intensity; CI: confidence interval, SD: standard deviation, IV: inverse variance; RMDQ: Roland Morris Disability Questionnaire Sources: [16-17,25]

Tanezumab 5 mg vs. tanezumab 10 mg: However, there was no significant difference between tanezumab 5 mg and tanezumab 10 mg in the reduction of RMDQ after 56 weeks [MD = -0.28 (-1.21, 0.66), P = 0.56]. The data were homogenous (P = 0.14, I2 = 54%). After 16 weeks, tanezumab 5 mg showed significantly less reduction of RMDQ compared to 10 mg [MD = 0.64 (0.11, 1.17), P = 0.02], and the data were homogenous (P = 0.82, I2 = 0), which is shown in Figure 8 and Table 4.

Adverse events

Patients with any adverse events

**Figure 9:**
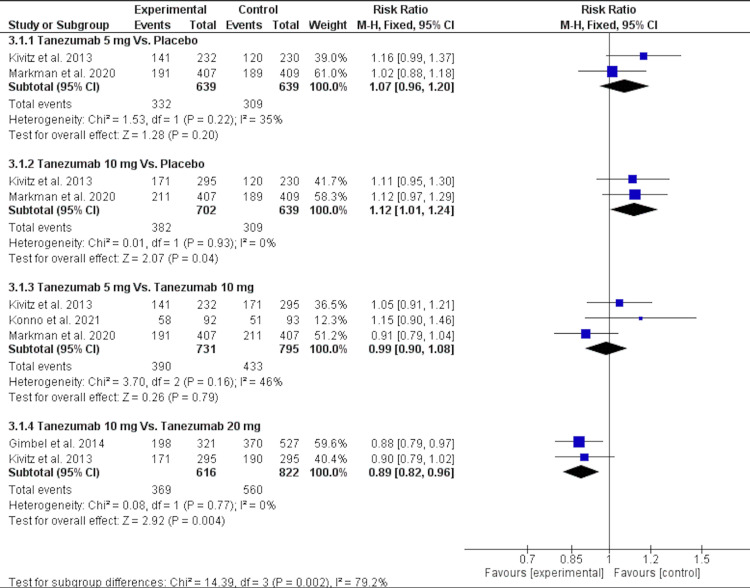
A forest plot of any adverse events outcomes CI: confidence interval, M-H: Mantel-Haenszel Sources: [16-17,25-26]

Concerning adverse events, there was no significant difference between tanezumab 5 mg over placebo and tanezumab 5 mg [RR = 1.07 (0.96, 1.2), P = 0.2]. The data were homogenous (P = 0.22, I2 = 35%). Compared to a placebo, tanezumab 10 mg showed a significantly higher incidence of adverse events [RR = 1.12 (1.01, 1.24), P = 0.04], and the data were homogenous (P = 0.93, I2 = 0). Moreover, tanezumab 20 mg had a substantially greater frequency of adverse events compared to tanezumab 10 mg [RR = 0.89 (0.82, 0.96), P = 0.004], and the data were homogenous (P = 0.77, I2 = 0), as shown in Figure 9 and Table 4.

Serious Adverse Events

**Figure 10:**
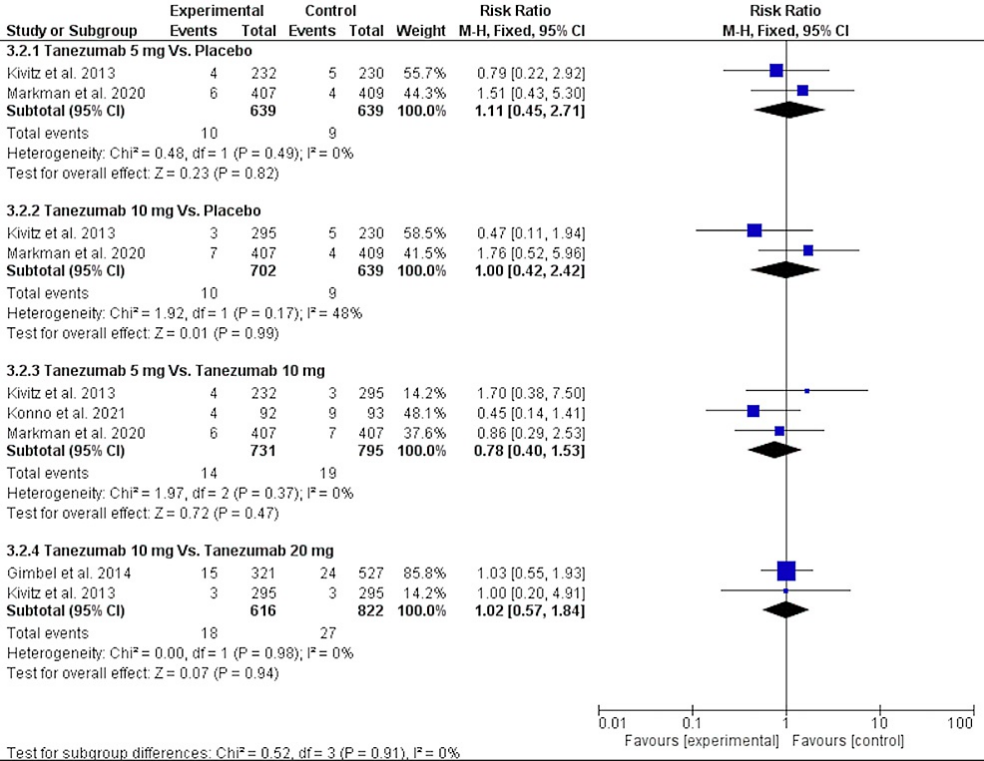
A forest plot of serious adverse events outcomes CI: confidence interval; M-H: Mantel-Haenszel Sources: [16-17,25-26]

Regarding serious adverse events, there was no significant difference between tanezumab 5 mg and placebo [RR = 1.11 (0.45, 2.71), P = 0.82]. The data were homogenous (P = 0.49, I2 = 0). There was no significant difference between tanezumab 10 mg and placebo [RR = 1.00 (0.42, 2.42), P = 0.99], and their data were homogenous (P = 0.17, I2 = 48%). There was no significant difference in serious adverse events between tanezumab 5 mg and tanezumab 10 mg [RR = 0.78 (0.40, 1.53), P = 0.47], and the data were homogenous (P = 0.37, I2 = 0). There was also no significant difference between tanezumab 20 mg and placebo [RR = 1.02 (0.57, 1.84), P = 0.94]. The data were homogenous (P = 0.98, I2 = 0), as evident in Figure 10 and Table 4.

Discontinuation due to Adverse Events

**Figure 11:**
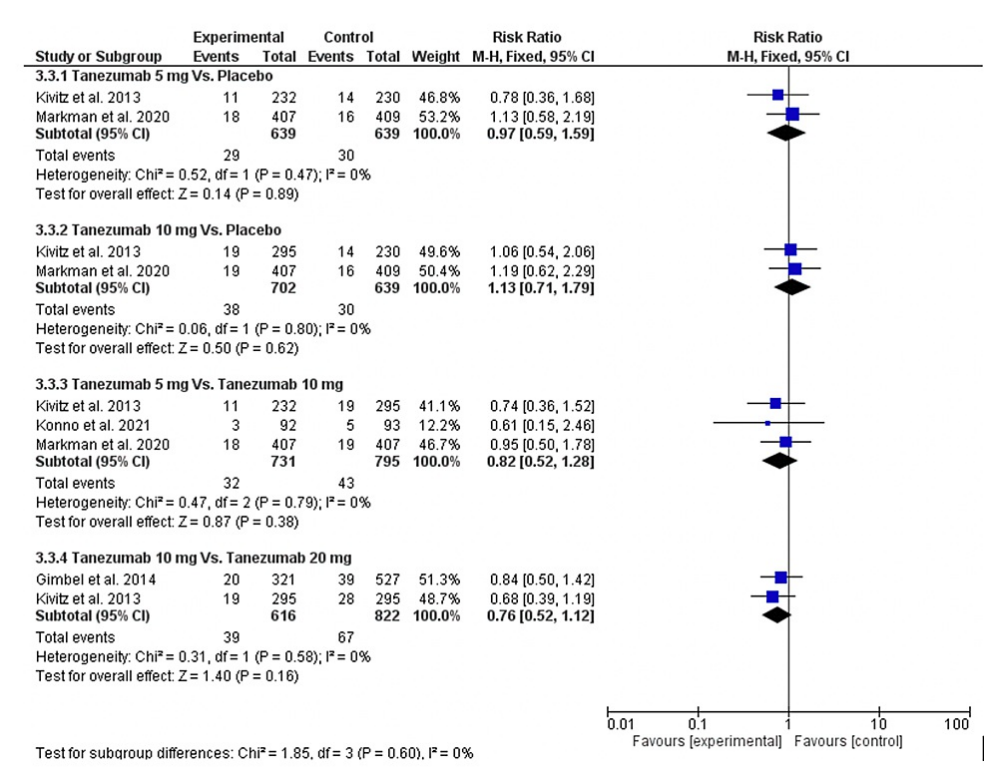
A forest plot of discontinuation due to adverse events CI: confidence interval; M-H: Mantel-Haenszel Sources: [16-17,25-26]

There was no significant difference in all the following comparisons: tanezumab 5 mg vs. placebo, tanezumab 10 mg vs. placebo, tanezumab 5 mg vs. tanezumab 10 mg, and tanezumab 10 mg vs. tanezumab 20 mg [RR = 0.97 (0.59, 1.59), P = 0.89], [RR = 1.13 (0.71, 1.79), P = 0.62], [RR = 0.82 (0.52, 1.28), P = 0.38], and [RR = 0.76 (0.52, 1.12), P = 0.16], respectively, as seen in Figure 11 and Table 4.

The data were homogenous (P = 0.47, I2 = 0), (P = 0.80, I2 = 0), (P = 0.79, I2 = 0), and (P = 0.58, I2 = 0), respectively.

Arthralgia

**Figure 12:**
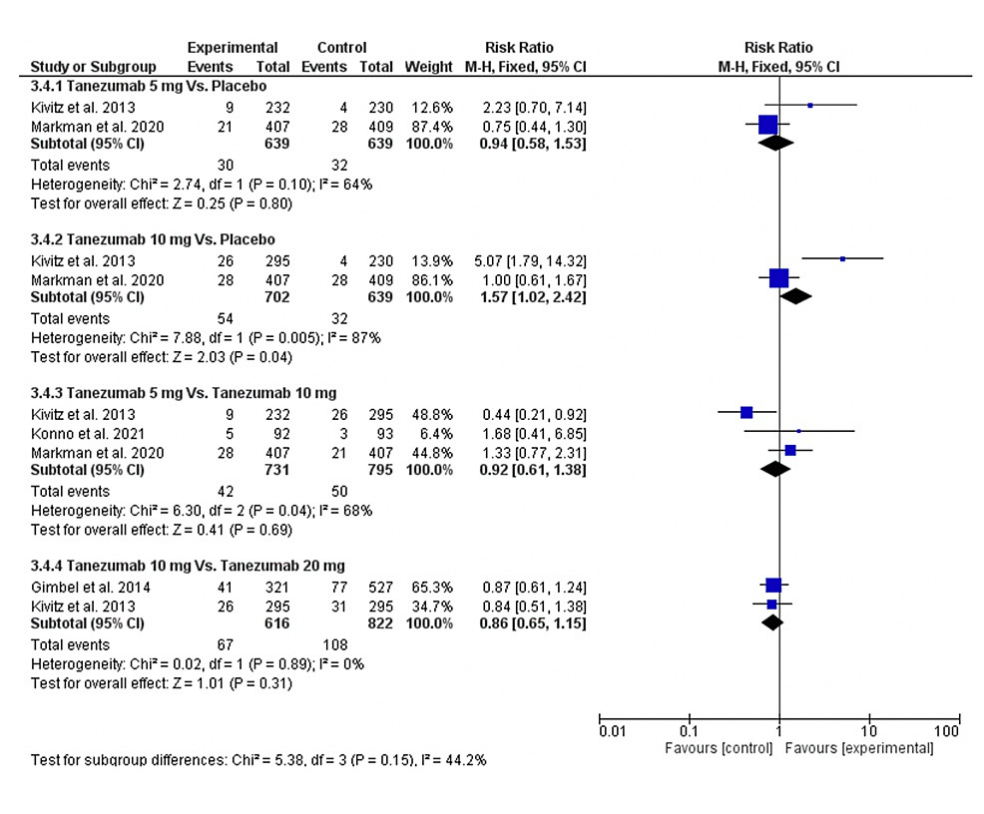
A forest plot of the arthralgia outcomes CI: confidence interval; M-H: Mantel-Haenszel Sources: [16-17,25-26]

Regarding the incidence of arthralgia, there was no statistically significant difference between tanezumab 5 mg and placebo [RR = 1.14 (0.41, 3.22), P = 0.8]. Still, the data were heterogeneous (P = 0.10, I2 = 64%), and this heterogeneity could not be resolved. Also, tanezumab 10 mg vs. tanezumab 20 mg showed no significant difference in the incidence of arthralgia [RR = 0.86 (0.65, 1.15), P = 0.31], and the data were homogenous (P = 0.89, I2 = 0) as shown in Figure 12 and Table 4.

Paresthesia

**Figure 13:**
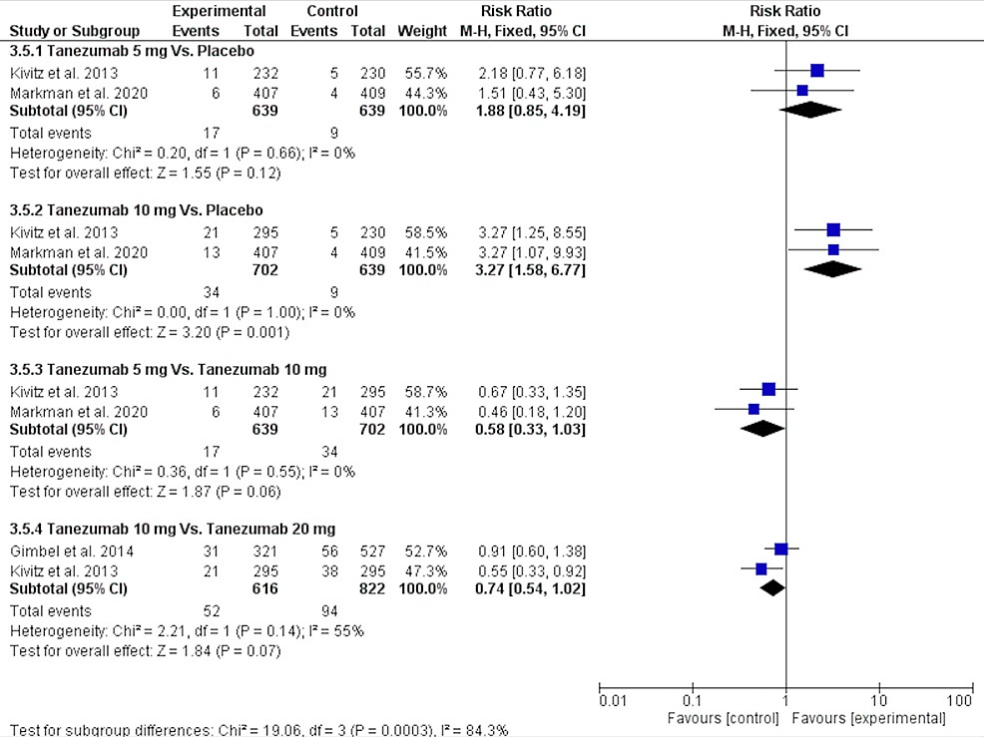
A forest plot of paresthesia outcomes CI: confidence interval; M-H: Mantel-Haenszel Sources: [16-17,25-26]

Regarding the incidence of paresthesia, there was no significant difference in the tanezumab 5 mg vs. placebo [RR = 1.88 (0.85, 4.19), P = 0.12], and the data were homogenous (P = 0.66, I2 = 0), as shown in Figure 13 and Table 4.

Tanezumab 10 mg, compared to placebo, showed a significantly higher incidence of paresthesia [RR = 3.27 (1.58, 6.77), P = 0.001], and the data were homogenous (P = 1.00, I2 = 0).

Discussion

The purpose of this systematic review and meta-analysis was to assess the effectiveness of tanezumab in the treatment of persistent low back pain. The six RCTs included in this study were conducted in diverse patient populations with varying chronic low back pain etiologies. Despite the heterogeneity in study populations, our analysis showed consistent results in the reduction of LBPI with tanezumab treatment. Our findings show that tanezumab 5 mg and 10 mg significantly reduced LBPI compared to placebo at all time points (two, four, eight, 12, and 16 weeks) examined. The effect size was larger for the 10 mg dose, consistent with previous studies examining the efficacy of tanezumab for chronic pain conditions.

Interestingly, our analysis also revealed that tanezumab 5 mg was significantly less effective than the 10 mg dose in reducing LBPI at two, four, eight, and 12 weeks. However, there was no significant difference between the two doses at 24, 32, and 40 weeks. This suggests that a higher dose of tanezumab may be required to achieve maximum pain reduction in the earlier stages of treatment. The study also compared the efficacy of tanezumab at 5 mg and 10 mg doses against a placebo in reducing RMDQ over 16 weeks. Both doses of tanezumab significantly reduced RMDQ compared to the placebo, with the 10 mg dose having a greater effect size. There was no significant difference in the reduction of RMDQ between the two doses over two to 56 weeks, except at 16 weeks, where the 10 mg dose showed a significantly greater reduction in RMDQ compared to the 5 mg dose.

Regarding adverse events, the results showed similarities between tanezumab 5 mg and placebo, but tanezumab 10 mg and 20 mg had a higher incidence of adverse events. There was no significant difference in the incidence of serious adverse events or patient discontinuation due to adverse events among the groups. The incidence of arthralgia and paresthesia was similar among the groups, except for tanezumab 10 mg vs. placebo, which showed a higher incidence of arthralgia. A previous meta-analysis by Lian et al. (2023) showed similar results [28]. They conducted a subgroup analysis according to the dose, but almost all subgroups only included one trial. We reported significant results regarding comparing doses of tanezumab with placebo and each other. We also provided more detailed safety outcomes and follow-up periods.

Several drugs can be used to manage chronic low back pain, each with its own pros and cons. One class of drugs commonly used are NSAIDs, such as ibuprofen and naproxen. These drugs effectively reduce pain and inflammation but can cause gastrointestinal problems, such as stomach ulcers and bleeding, especially when used for long periods [29]. Another class of drugs is opioids, such as oxycodone and hydrocodone. Opioids can provide effective short-term pain relief, often leading to dose escalation, thus carrying a high risk of addiction and overdose. They can also cause side effects such as constipation, dizziness, and nausea [9,30]. Antidepressants, such as duloxetine and amitriptyline, can also be used to treat chronic low back pain. They alter the brain's perception of pain but can cause side effects such as drowsiness, dry mouth, and blurred vision [31]. Finally, muscle relaxants such as cyclobenzaprine can relieve muscle spasms that can contribute to low back pain. However, they can cause drowsiness and dizziness and may interact with other medications [32].

This study has several strengths, as we employed a thorough search strategy across multiple databases to identify relevant studies. The primary outcome was clearly defined, and the results were reported in a standardized manner. The meta-analysis demonstrated homogeneity in most comparisons, indicating consistency of results across the included studies. We analyzed the data after up to 56 weeks and compared the different doses of tanezumab with placebo and each other. However, there are also some limitations to the study. The number of studies included in the meta-analysis is relatively small, which may limit the generalizability of the findings. Most of the included studies were conducted in developed countries with a predominantly white population, which may limit the generalizability of the findings to other populations. Three studies were deemed to have a high risk of bias in the domain of missing outcome data, which may impact the validity of the results.

## Conclusions

In conclusion, tanezumab significantly reduced LBPI compared to placebo over various time intervals. Furthermore, tanezumab 10 mg exhibited superior efficacy in reducing low back pain intensity at 16 weeks compared to tanezumab 5 mg. Tanezumab reduced disability (RMDQ) scores at 16 weeks, and adverse events were comparable among groups, with tanezumab 10 mg showing a higher incidence than placebo. Tanezumab 10 mg was associated with a significantly increased risk of paraesthesia compared to placebo. Overall, the study suggests that tanezumab can effectively relieve low back pain, but careful consideration of dose-related adverse events is warranted.
